# Label-Free Imaging of Umbilical Cord Tissue Morphology and Explant-Derived Cells

**DOI:** 10.1155/2016/5457132

**Published:** 2016-09-26

**Authors:** Raf Donders, Kathleen Sanen, Rik Paesen, Eli Slenders, Wilfried Gyselaers, Piet Stinissen, Marcel Ameloot, Niels Hellings

**Affiliations:** ^1^Biomedical Research Institute, Hasselt University and School of Life Sciences, Transnational University Limburg, Agoralaan Building C, 3590 Diepenbeek, Belgium; ^2^Ziekenhuis Oost-Limburg, Campus St. Jan, Schiepse Bos 6, 3600 Genk, Belgium

## Abstract

*In situ* detection of MSCs remains difficult and warrants additional methods to aid with their characterization* in vivo*. Two-photon confocal laser scanning microscopy (TPM) and second harmonic generation (SHG) could fill this gap. Both techniques enable the detection of cells and extracellular structures, based on intrinsic properties of the specific tissue and intracellular molecules under optical irradiation. TPM imaging and SHG imaging have been used for label-free monitoring of stem cells differentiation, assessment of their behavior in biocompatible scaffolds, and even cell tracking* in vivo*. In this study, we show that TPM and SHG can accurately depict the umbilical cord architecture and visualize individual cells both* in situ* and during culture initiation, without the use of exogenously applied labels. In combination with nuclear DNA staining, we observed a variance in fluorescent intensity in the vessel walls. In addition, antibody staining showed differences in Oct4, *α*SMA, vimentin, and ALDH1A1 expression* in situ*, indicating functional differences among the umbilical cord cell populations. In future research, marker-free imaging can be of great added value to the current antigen-based staining methods for describing tissue structures and for the identification of progenitor cells in their tissue of origin.

## 1. Introduction

Stem cells originating from perinatal tissues such as the umbilical cord (UC) are being intensively studied for application in regenerative medicine. Due to their intrinsic growth promoting abilities mediated via self-renewal, multilineage differentiation, and trophic factor production, as well as their immune modulatory functions [1, 2], these perinatal stem cells are put forward as potent alternatives to adult stem cell sources for both autologous and allogeneic application. As a result, UC-derived stromal cells are currently under evaluation as cellular therapy for multiple degenerative diseases and as an immune modulatory approach for diseases involving aberrant immunological responses, such as multiple sclerosis, Parkinson's disease, graft-versus-host disease, type 1 diabetes, or stroke [3–6].

The UC is a rich source of stem cells, since a variety of progenitors can be harvested from different compartments of the tissue, for example, cord blood, perivascular space, and tissue matrix [7, 8]. The derivation of multipotent cells from the UC matrix or Wharton's jelly (WJ) was first described about a decade ago by Mitchell et al. and Romanov et al., reporting the isolation of stromal cells with a mesenchymal-like phenotype (WJ-MSCs) [9, 10]. Following their discovery, their potent preclinical potential as well as their superior culture properties over adult (bone-marrow-derived) mesenchymal stem cells (MSCs) has been extensively described [11–16]. In contrast to this extensive characterization in culture, the biology of WJ-MSCs* in situ* and their transition from tissue into culture remain poorly understood.

At present, the identification of mesenchymal-like cells derived from a specific tissue of origin relies on* in vitro* assays which usually involve the dissociation of the tissue and isolation and culturing of cells first. Classically, MSCs are defined by the ability to adhere to plastic, the expression of specific surface marker antigens, and multipotent differentiation potential [17]. To assess cell differentiation and function at specific time points or within a certain tissue, techniques such as western blots, quantitative polymerase chain reaction, and immunohistochemistry are the most commonly utilized. Although these approaches are highly sensitive and specific, their destructive nature does not allow for dynamic or real-time assessments of cells within intact tissues [18]. As such, the* in situ *identification of MSCs remains difficult and requires additional imaging methods.

Nonlinear optical microscopy techniques, such as multiphoton microscopy and higher harmonic generation, are emerging tools for intravital noninvasive imaging of cells and tissues [19–21]. These techniques allow for marker-free visualization and characterization of cells and tissue structures without fixation or staining procedures [19, 22]. Accordingly, two-photon excitation can provoke the emission of photons from intrinsic fluorophores within the cell, such as nicotinamide adenine dinucleotides (NADH) and flavins, a phenomenon called autofluorescence (AF) [23, 24]. In addition, asymmetric molecules such as collagen type I and elastin can produce light at exactly twice the frequency (or half the wavelength) of the pulsed excitation beam, a feature that is referred to as second harmonic generation (SHG) [21, 25]. SHG does not suffer from photobleaching and allows for extended periods of observation [26]. Moreover, the two-photon laser excitation beams can penetrate deeper into the tissue allowing imaging and tracking of cells in relatively thick samples of up to 1 mm [20, 27, 28]. By monitoring AF and SHG, stem cell differentiation, cell behavior in 3D biological scaffolds (e.g., collagen matrices), and* in vivo* tracking of cells (untouched or transgenic) and regenerative processes have been visualized [28–37].

The aim of the present study is to assess the potential of label-free imaging for the visualization of cells within umbilical cord tissue and for monitoring stromal cells during explant isolation and in culture. Our data show that two-photon fluorescence microscopy (TPM) and SHG imaging can be used to detect cells* in situ* without exogenously applied labeling molecules. We were able to visualize the UC architecture along with explant attachment and primary cell outgrowth. In parallel, chondrogenic pellets were imaged to validate the procedure, showing collagen rich deposits and cells in cleft-like structures after differentiation of WJ-MSCs. Furthermore, AF and SHG imaging was used in combination with nuclear DNA staining, revealing differential intensities in nuclear fluorescence in the umbilical vessel walls. As such, we show that TPM is an elegant tool to characterize UC stem cells* in situ*, with the potential for parallel use with conventional imaging and staining techniques.

## 2. Material and Methods

### 2.1. Umbilical Cord Tissue Processing

The collection and experimental use of human UC tissues were approved by the Medical Ethical Committees of Hasselt University and Ziekenhuis Oost-Limburg. UC tissues (*n* = 5) were obtained aseptically from full-term uncomplicated pregnancies with planned cesarean section, after informed consent. Cords were drained of blood and subsequently stored in sterile phosphate-buffered saline (PBS; Lonza, Verviers, Belgium) supplemented with 1% penicillin-streptomycin (P/S; 10000 : 10000 U; Gibco®, Life Technologies, Gent, Belgium) and 0.2% Fungizone® (250 *μ*g/mL; Gibco, Life Technologies). Tissues were processed within 24 hours for cell isolation or sectioning. Fresh cord fragments were processed for cell isolation (see below) or fixed overnight with 4% paraformaldehyde (PFA; Sigma-Aldrich, Bornem, Belgium) followed by paraffin embedding. For subsequent* in situ* analysis, 7 *μ*m sections were deparaffinized in xylene (VWR, Heverlee, Belgium) and rehydrated in graded ethanol series until submerged in PBS.

### 2.2. Wharton's Jelly Stem Cell Culture

Stromal cells were isolated from the WJ using explant tissue culturing as was previously described [4]. In brief, after removal of the vessels, the cord matrix was cut into 2 mm^3^ fragments and cultured in KnockOut*™* Dulbecco's modified Eagle's medium with F12 (Gibco, Life Technologies) supplemented with 1% P/S, 1% GlutaMAX*™* (200 mM; Gibco, Life Technologies), and 10% fetal bovine serum (Biochrom AG, Berlin, Germany). When cellular outgrowth from the explants was observed, fresh medium was added every 3 days. For imaging, explants were seeded in 8-well chamber slides (*μ*-slide, Ibidi, Martinsried, Germany). Wharton's jelly-derived stem cells (WJ-MSCs) were collected at 80% confluence using StemPro® accutase (Gibco, Life Technologies) and seeded either in T75 flasks (Nunc*™*; VWR) for further expansion, on glass coverslips (Menzel-Gläser; Braunschweig, Germany) for characterization experiments, or in 8-well chamber slides for TPM and SHG imaging.

### 2.3. Trilineage Differentiation

Differentiation was performed as previously described [38], using the human Mesenchymal Stem Cell Functional Identification Kit (SC006; R&D Systems, Abingdon, Oxfordshire, UK). For adipogenic and osteogenic differentiation, WJ-MSCs were cultured for 3 weeks in 24-well plates (Nunc) on sterile glass coverslips, in their respective complete differentiation medium according to the kit instructions. Medium was changed every 3 days after which cells were fixed with 4% PFA and stored in PBS at 4°C until microscopy imaging. To validate the monolayer differentiation cultures, adipogenic coverslips were stained with Oil Red O (ORO; Sigma-Aldrich) as previously described [38], whereas osteogenic coverslips were stained with the anti-osteocalcin antibody from the differentiation kit. For chondrogenic differentiation, freshly harvested WJ-MSCs were transferred to 15 mL conical tubes containing 0.5 mL complete chondrogenic differentiation medium. After centrifugation, pellets were cultured for 3 weeks with medium changed every 3 days. After that, chondrogenic pellets were fixed with 4% PFA and stored in PBS at 4°C until microscopic imaging and Alcian Blue staining. For multiphoton microscopy, whole chondropellets were submerged in PBS in chamber slides. To further validate the differentiation process, pellets were snap frozen and sectioned into 7 *μ*m tissue slices using a Leica CM1900UV cryostat (Leica Microsystems, Diegem, Belgium). Thawed cryosections were hydrated with distilled water and stained with Alcian Blue (generated in-house) for 30 minutes in the dark. Next, the sections were washed and counterstained with Nuclear Fast Red (NFR; Sigma-Aldrich) for 5 minutes, dehydrated, and mounted with glass coverslips using DPX (Merck, Darmstadt, Germany). Bright field images were taken using a Nikon Eclipse 80i microscope and processed with NIS Elements BR 4.0 software (Nikon Instruments BeLux, Brussels, Belgium).

### 2.4. Multiphoton Microscopy and SHG Imaging

AF and SHG imaging of the UC tissue slices, explants, and stem cells was performed with a Zeiss LSM 510 META mounted on an Axiovert 200 M (Carl Zeiss, Jena, Germany) and equipped with a femtosecond pulsed laser excitation source (Mai Tai DeepSee, Spectra-Physics, CA, USA) tuned to a central wavelength of 810 nm. For scanning an entire UC section, a 10x/0.3 objective (Plan-Neofluar 10x/0.3, Carl Zeiss) was used. Detailed images were taken through a 40x/1.1 water immersion objective (LD C-Apochromat 40x/1.1 W Korr UV-VIS-IR, Carl Zeiss). As a control for cellular location, UC tissue slices (*n* = 2) were counterstained with 0.1% 4′,6-diamidino-2-phenylindole (DAPI; 1 mg/mL; Molecular Probes®, Life Technologies) in distilled water. For imaging the explant process, WJ-MSCs in culture, or the chondrogenic pellets, a 20x/0.75 objective was selected (Plan-Apochromat 20x/0.75, Carl Zeiss). Both AF and SHG were detected in backward nondescanned mode by analogue photomultipliers. The signals were first separated from the excitation beam using a long pass dichroic mirror with an edge at 685 nm. Next, the SHG and AF were separated from each other by a long pass dichroic mirror with an edge at 442 nm. The SHG signal then passed through a 10 nm narrow band pass filter with a central wavelength of 405 nm. In the AF channel, a wide band pass filter ranging from 450 nm to 650 nm was used to clean out any possible leaked excitation and SHG light. 3D images were obtained after digitally combining Z-stack optical sections. All images were processed using ZEN 2009 Light Edition software (Carl Zeiss).

### 2.5. Immunohistochemistry

For marker expression analysis, 7 *μ*m thick tissue slices were microwaved for antigen retrieval in 10 mM sodium citrate buffer at pH 6.0 (Sigma-Aldrich). Next, specific antigen expression was detected using the peroxidase-based EnVision*™*+ system (Dako, Heverlee, Belgium) according to the manufacturer's instructions. Prior to labeling with the primary antibody, tissues were permeabilized in Tris-buffered saline (VWR) with 0.05% Tween-20 (Merck Chemicals, Overijse, Belgium) (TBS-T), after which endogenous peroxidase activity was quenched with 0.5% hydrogen peroxide. Nonspecific binding sites were blocked using 10% normal goat serum (Dako) in TBS-T. Subsequently, tissues were incubated for 2 hours in TBS-T with primary antibodies directed against human octamer-binding transcription factor 4 (Oct4; 1/250; rabbit polyclonal ab19857; Abcam, Cambridge, UK), aldehyde dehydrogenase family 1 member A1 (ALDH1A1; 1 *μ*g/mL; rabbit polyclonal ab23375; Abcam), alpha smooth muscle actin (*α*SMA; 1/50; mouse monoclonal *α*sm-1; Novocastra*™*, Leica), vimentin (1/100; mouse monoclonal V9; Dako), and pan cytokeratin (pan-CK; 1/100; mouse monoclonal MNF116; Dako), followed by 30-minute incubation with horseradish peroxidase- (HRP-) conjugated goat anti-rabbit or goat anti-mouse secondary antibody (Envision kit). To visualize binding of the antibodies, diaminobenzidine (DAB) chromogen substrate was added after which the tissues were counterstained with Mayer's hematoxylin (Leica) or NFR in case of nuclear antigen detection. Stainings without primary antibody served as negative controls. Next, stained sections were dehydrated and mounted with glass coverslips using DPX. Sections were examined using a Mirax Desk photomicroscope slide scanner and images were processed with Mirax Viewer software (Carl Zeiss).

### 2.6. Immunocytochemistry

WJ-MSCs were seeded on sterile glass coverslips in a 24-well plate and grown until 80% confluence. WJ cells were fixed with 4% PFA before antibody staining with the EnVision*™*+ system. The cell membrane was permeated and nonspecific binding sites were blocked using PBS supplemented with 0.3% Triton X-100 (Sigma-Aldrich), 1% bovine serum albumin (US Biological, Swampscott, MA, USA), and 10% normal goat serum (blocking buffer) at room temperature for 45 minutes. Next, the cells were incubated for 2 hours with 5 *μ*g/mL Oct4 (ab19857; Abcam), 1 *μ*g/mL ALDH1A1 (ab23375; Abcam), or *α*SMA (1/100, *α*sm-1; Novocastra, Leica) in blocking buffer. The negative controls were incubated without the primary antibody. Subsequently, the coverslips were washed and incubated for 1 hour with HRP-conjugated goat anti-rabbit or goat anti-mouse secondary antibody of the Envision kit. After washing the cells, nuclear counterstaining with Mayer's hematoxylin or NFR was performed, and coverslips were subsequently mounted on glass slides using Aquatex (Merck). Stained cells were examined using a Nikon Eclipse 80i microscope and images processed with NIS Elements BR 4.0 software (Nikon Instruments BeLux, Brussels, Belgium).

## 3. Results

### 3.1. Detailed Visualization of Different Anatomical Compartments within the Umbilical Cord Using TPM and SHG Detection

**Figure 1:**
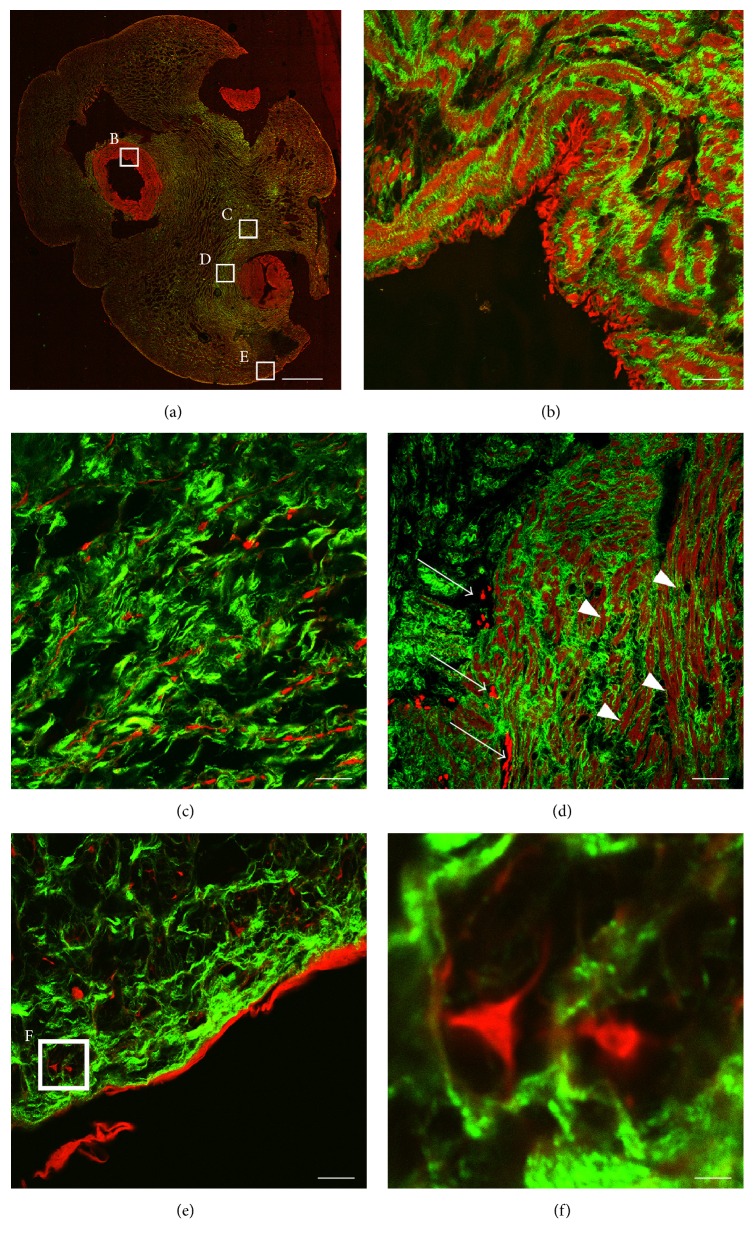
Label-free imaging of umbilical cord tissue compartments. (a) Image composition derived from multiple fields of view for AF (red) and SHG (green) in unstained cord tissue, scale bar = 1 mm. (B–E) Detailed image sections of (a), scale bars = 20 *μ*m. (b) Lamina intima and media of the umbilical vein. (c) Wharton's jelly and perivascular zone of an umbilical artery. (d) Umbilical artery media and adventitia showing intensely fluorescent cells (arrows) and less bright smooth muscle cell bodies (arrowheads). (e) Subamnion and amniotic epithelial layer. (F) Detailed image of (e) displaying tripolar cells within the subamniotic zone, scale bar = 2 *μ*m. Representative images from 3 independent donors are shown.

Based on AF and SHG signals, a detailed image of the UC architecture was generated.  Figure 1(a) shows a composition of serial scanned sections of cord tissue after TPM imaging. Without any additional labeling agents, we clearly observed the overall cord composition and the cellular organization of the umbilical cord vessels, WJ, and subamniotic zone. The UC is mainly composed of a gelatinous matrix of different types of collagen supporting the two arteries and vein [39]. We detected the different tissue layers of the umbilical vein and arteries, as is depicted in Figure 1(a) and detailed in Figures 1(b) and 1(d). Within the stromal clefts lining the vessels, slender myofibroblast-like cells were observed (Figures 1(c) and 1(d)). Furthermore, we detected the presence of tripolar-shaped cells within the WJ and subamniotic region (Figures 1(c), 1(e), and 1(f)). Finally, some hollow areas surrounding the vessels were observed (Figure 1(a)), likely caused by the presence of extraluminal blood that was washed out during sectioning.

### 3.2. Cellular Autofluorescence Does Not Discriminate between Stromal Cells but Is Less Bright in Vascular Smooth Muscle Cells

**Figure 2:**
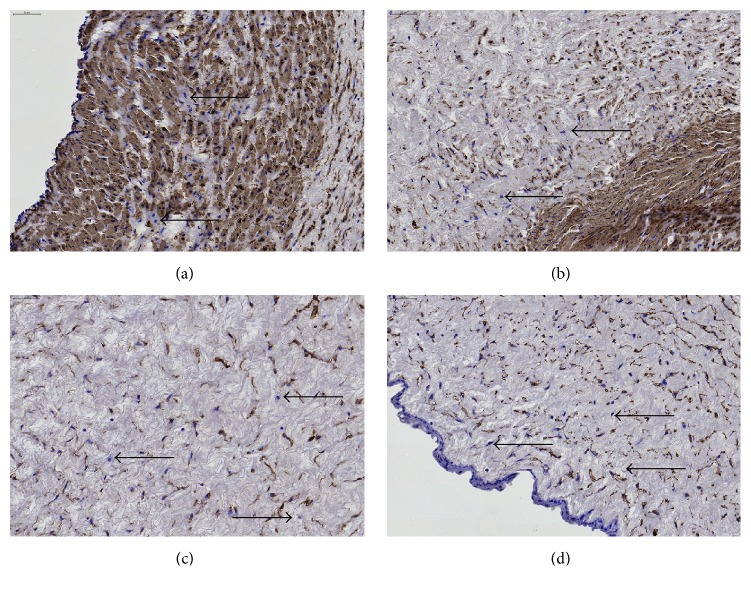
Staining of umbilical cord tissue for *α*SMA expression (brown). Images are shown for umbilical cord areas: (a) vein, (b) arteries, (c) Wharton's jelly, and (d) cord edge and amniotic epithelium. Slides were counterstained using Mayer's hematoxylin (dark blue = cell nuclei). Scale bars = 50 *μ*m. Unstained cells (black arrows) are found in all anatomical compartments of the cord. Representative images of 3 different experiments are shown. Images of control staining without primary antibody are available in the electronic Supplementary Material, Figure  S1, available online at http://dx.doi.org/10.1155/2016/5457132. Scale bars = 50 *μ*m (top left corner).

**Figure 3:**
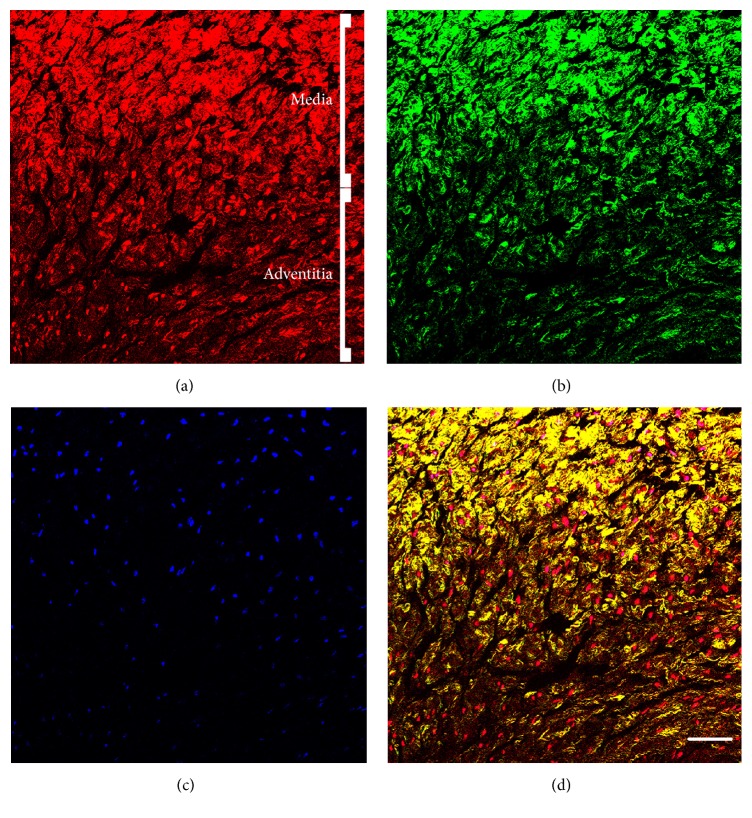
TPM and SHG imaging of DAPI stained umbilical cord vein showing a differential staining intensity for DAPI. (a) AF signal and (b) SHG derived from the vessel media and adventitia. Increased AF density originates from the media smooth muscle cells. In addition, more SHG is observed in the media due to higher collagen content, as expected. (c) An increasing gradient in DAPI fluorescence was observed towards the media of the umbilical vein. (d) Merged image. Scale bar = 50 *μ*m.

Autofluorescence was observed throughout the entire umbilical cord and was more localized to the vessel walls (Figure 1). This fluorescence signal originated from the highly abundant smooth muscle cells residing in the collagen deposits of the media, as was shown in parallel by *α*SMA staining (Figure 2 and Figure  S1). Although autofluorescence was more confined to the vessels due to higher cell numbers, it was observed that the signal intensity of the surrounding stromal cells was higher compared to that of the smooth muscle fibers (Figure 1(d)). Nevertheless, no major difference in autofluorescence was observed between the stromal cells populations residing in the other compartments (Figure 1). Additionally, the vessels were further examined for cellular presence by nuclear staining with DAPI. Surprisingly, we observed a variance in nuclear fluorescent signal intensity amongst the cells residing in different areas of the vessel wall. As seen in Figure 3, cells located in the smooth muscle and collagen rich media of the vessel wall (red and green fluorescence, resp., Figures 3(a) and 3(b)) show higher nuclear fluorescence intensity compared to cells from the perivascular adventitia (Figure 3(c)).

### 3.3. Imaging of Live WJ Explants and Cellular Outgrowth

**Figure 4:**
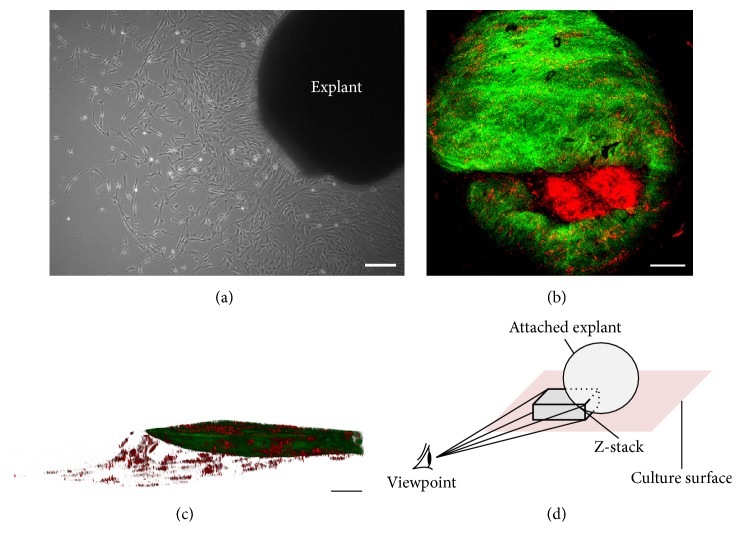
Marker-free visualization of Wharton's jelly explant tissue and cell outgrowth. (a) Phase contrast image of an attached explant with outgrowing WJ-MSCs, scale bar = 200 *μ*m. (b) AF (red) and SHG (green) of an attached explant (attachment site, bottom view), scale bar = 100 *μ*m. (c) 2D image of cellular outgrowth from explant tissue at the attachment site (side view), scale bar = 50 *μ*m. The images were derived from a Z-stack composition of the SHG and AF signals of (b). (d) Schematic view of the visualization plane of (c). Representative images for 3 independent experiments are shown.

Attached WJ explants were obtained approximately 10 days after isolation and subsequently imaged with TPM. In a label-free manner, we were able to visualize both the attached tissue structure and the outgrowing cells (Figure 4(b)). Our observation correlated with the cellular outgrowth visualized by bright field microscopy (Figure 4(a)). By scanning the explant attachment area (Z-stack; Figures 4(c) and 4(d)), we found a cellular migration pattern, showing a sloped downward outgrowth from the globular explant to the culture surface.

### 3.4. Validation of WJ-MSCs Chondrogenic Differentiation

**Figure 5:**
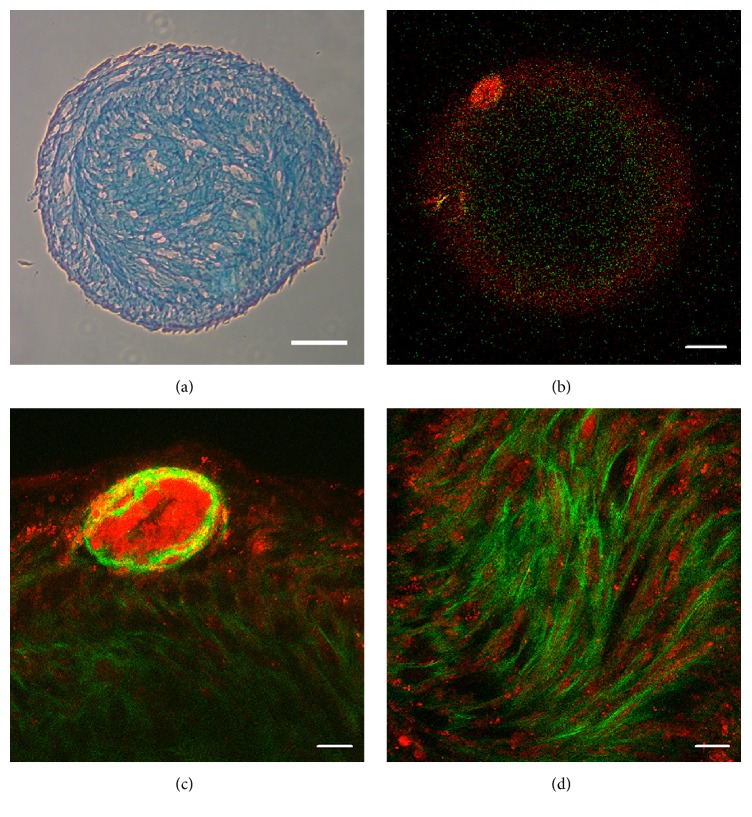
TPM and SHG imaging of WJ-MSCs chondrogenic differentiation. (a) Alcian Blue staining of a chondrogenic pellet section, visualizing the nuclei (red) and chondrogenic matrix (blue), scale bar = 200 *μ*m. (b–d) AF (red) and SHG (green) imaging of an intact chondrogenic pellet of ±1 mm diameter. (b) Chondrogenic pellet center, scale bar = 100 *μ*m. (c) Cell and matrix nodule at the pellet border, scale bar = 20 *μ*m. (d) Differentiated cells within their collagen rich matrix in the center of the pellet, scale bar = 20 *μ*m. Representative images for 2 independent experiments are shown.

To confirm the validity of our imaging approach, chondrogenic differentiation of WJ-MSCs was assessed. Detection of SHG after stem cell differentiation has already been reported for adult stromal cells [40, 41]. The chondrogenic pellet consists of a complex matrix containing glycosaminoglycans, collagen, and proteoglycans with stromal cells scattered throughout the matrix scaffold, as was shown in Figure 5(a) by Alcian Blue staining. Indeed, based on their autofluorescence, WJ-MSCs were observed in the pellet's cleft-like structures visualized by SHG (Figures 5(c) and 5(d)). Furthermore, a bright nodule composed of a cellular center surrounded by collagen could be observed at the edge of the pellet (Figures 5(b) and 5(c)). In addition, we imaged the adipogenic and osteogenic differentiation end state (Figure  S3). After adipogenic differentiation (Figures  S3a and S3c), confirmed by ORO staining, we observed the typical voids in fluorescent signal due to lipid droplet accumulation. For the osteogenic differentiation (Figures  S3b and S3d), low SHG signal was detected, originating from collagen deposition in the ECM during differentiation. Validity of the differentiation was shown by* de novo* osteocalcin expression.

### 3.5. TPM Imaging of WJ-MSCs in Culture Indicates That Autofluorescence Originates from the Perinuclear Organelles

**Figure 6:**
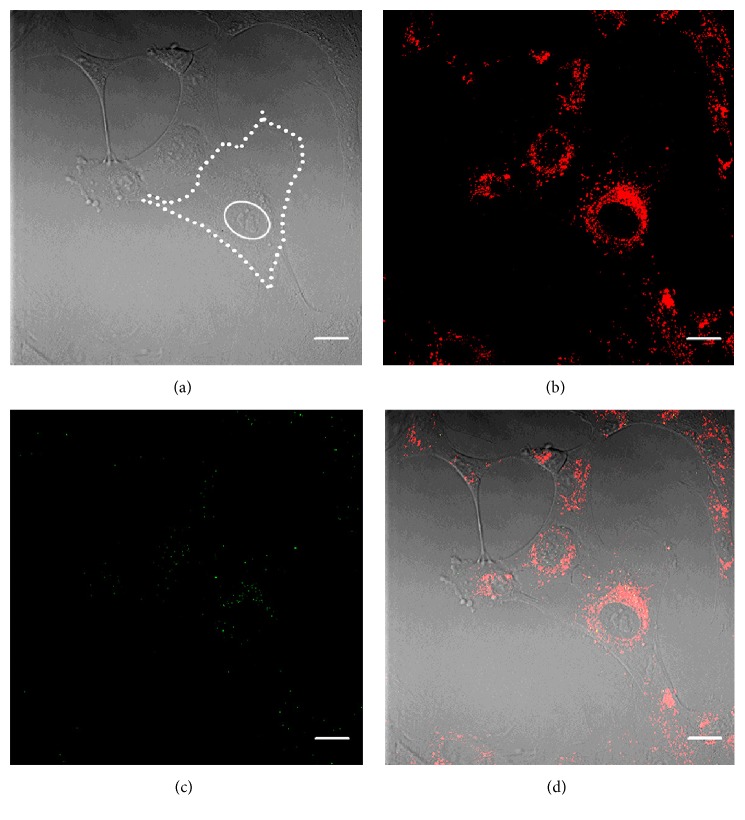
Autofluorescence originates from the perinuclear organelles in WJ-MSCs. (a) Bright field image of explant-derived WJ-MSCs. For reference, the cell boundary is delineated by a dotted white line and the nucleus is outlined by a full white line. (b) AF signal (red) and (c) SHG after two-photon excitation of the same cells. (d) Merged image. A perinuclear area of organelles is visible (view also the electronic Supplementary Material, Figure  S2). Scale bars = 20 *μ*m.

Besides umbilical cord tissue, cultures of explant-derived cells were also visualized using TPM. As shown in Figure  S2, WJ-MSCs have a fibroblast-like morphology and possess large nuclei and multiple nucleoli and have their organelles confined to the perinuclear region of the cytoplasm. The latter was also shown by Struys et al. at the ultrastructural level [38]. Interestingly, in this study, we observed autofluorescence originating from the perinuclear zone of live cells in culture (Figures 6(b) and 6(d)). Few SHG scatter could be observed (Figure 6(c)).

### 3.6. Differences in Oct4, *α*SMA, Vimentin, Pan-CK, and ALDH1A1 Expression* In Situ*

**Figure 7:**
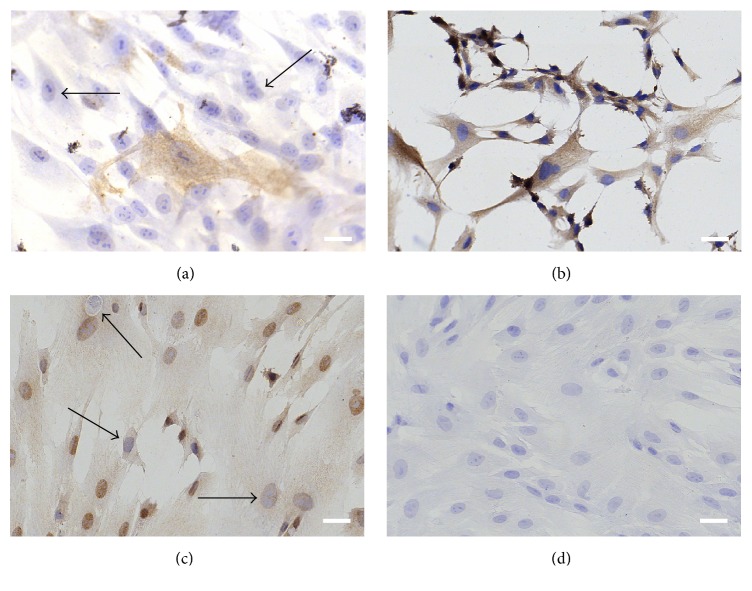
Staining of WJ-MSCs in culture for (a) *α*SMA, (b) ALDH1A1, and (c) Oct4 expression. (d) Representative control staining without primary antibody. All cells stain positive (brown) for ALDH1A1, but not for *α*SMA and Oct4 (black arrows), indicating that a heterogeneous cell isolate was obtained. Scale bars = 50 *μ*m. Representative images of at least 3 donors are shown.

**Figure 8 fig8:**
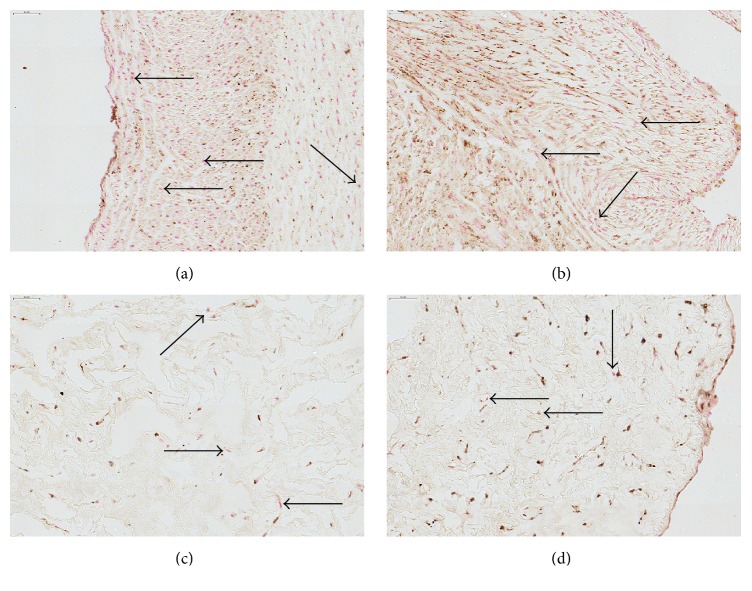
Staining of umbilical cord tissue for Oct4 expression (brown). Nuclei (red) were visualized using NFR counterstain. Images are shown for umbilical cord areas: (a) vein, (b) arteries, (c) Wharton's jelly, and (d) cord edge and amniotic epithelium. Scale bars = 50 *μ*m. Unstained cells (black arrows) are found in all anatomical compartments of the cord. Representative images of 3 different experiments are shown. Images of control staining without primary antibody are available in the electronic Supplementary Material, Figure  S1. Scale bars = 50 *μ*m (top left corner).

**Figure 9:**
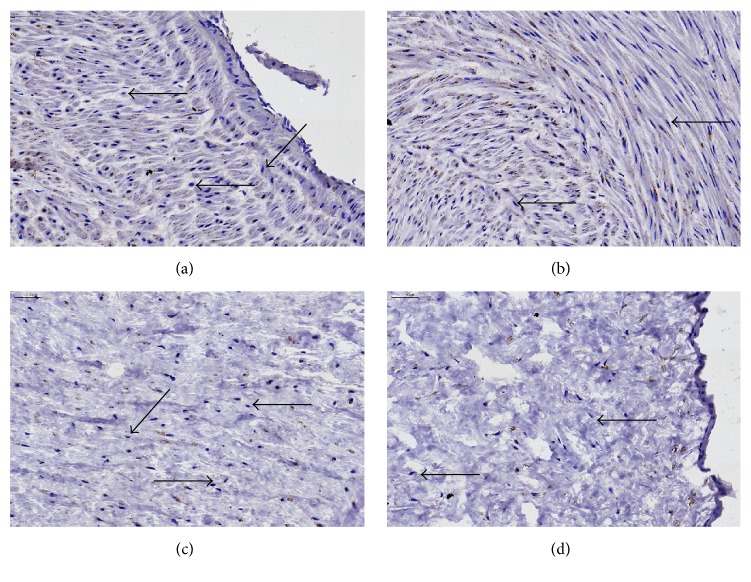
Staining of umbilical cord tissue for ALDH1A1 expression (brown). Images are shown for umbilical cord areas: (a) vein, (b) arteries, (c) Wharton's jelly, and (d) cord edge and amniotic epithelium. Slides were counterstained using Mayer's hematoxylin (dark blue = cell nuclei). Scale bars = 50 *μ*m. Unstained cells (black arrows) are found in all anatomical compartments of the cord. Representative images of 3 different experiments are shown. Images of control staining without primary antibody are available in the electronic Supplementary Material, Figure  S1. Scale bars = 50 *μ*m (top left corner).

As was previously shown by us and others, cultured WJ-MSCs express several surface and intracellular markers, such as the classical MSCs phenotype panel, but also several other, for instance, multipotency markers (e.g., Oct4, nucleus), adhesion molecules (e.g., CD54, membrane), or immune modulatory molecules (e.g., IDO-1, cytosol) [4, 42, 43]. Here, we attempted to localize the WJ-MSCs* in situ* by assessing the expression of Oct4, a transcription factor related to the pluripotent stem cell state [44]. In addition, we performed stainings for *α*SMA, in order to assess the perivascular niche of smooth muscle and myofibroblast-like cells, and ALDH1A1 which is expressed in various stem cell populations [45]. Furthermore, we assessed the expression of cytoskeletal proteins by staining for pan-CK and vimentin (an intermediate filament found in cells of mesenchymal origin) [38, 46]. We could not correlate a specific expression pattern for these markers to a particular anatomical location within the umbilical cord, as both positive and negative cells were found in all areas (Figures 7 [Fig fig8]–9 and Figure  S4). Respective negative control stainings are depicted in the electronic Supplementary Material, Figures  S1, S4c, S4f, S4i, and S4l.

*α*SMA expression was intensely observed in the umbilical vessels, as was to be expected because of the smooth muscle cells presented there. Interestingly, also WJ stromal cells expressed *α*SMA (Figures 7(a) and 2); however, we could not observe increased cellular staining within the perivascular zone compared to other areas (data not shown). Analysis of vimentin and pan-CK expression indicated that positive cells are scattered throughout the entire umbilical cord, including the perivascular areas (Figures  S4a–S4f), stroma (Figures  S4g–S4i), and subamniotic zone (Figures  S4j–S4l). Of note, the cord lining epithelial membrane did not express vimentin* in situ* but showed intense pan-CK staining. Oct4 was expressed in cultured WJ cells (Figure 7(c)) and also* in situ* by most perivascular cells, stromal cells, and even amniotic epithelial cells (Figure 8). ALDH1A1 staining is more confined to the media of the umbilical vessels, but also WJ matrix cells express the protein (Figure 9). Interestingly, in WJ cell cultures, all cells were positive for ALDH1A1 (Figure 7(b)), indicating that either a specific cell population is isolated into culture or a culture induced expression occurs. Moreover, we observed a variable expression intensity within the heterogeneous culture, where mainly the smaller cells displayed a darker staining pattern.

## 4. Discussion

Multiphoton and higher harmonic generation imaging offers a high-resolution characterization tool for tissues and stem cells, because of its noninvasive and marker-free nature, whereas traditional assessments of tissue structure are destructive at both the molecular and the structural level (e.g., protein expression and extracellular matrix degradation). Consequently, this nondestructive, label-free approach offers a powerful high-content characterization tool for optimizing tissue engineering protocols and assessing engineered tissue implants [47]. Furthermore, the possibility for noninvasive optical tracking of cells and tissue structure* in vitro* can be applied in future studies to assess tissue development, drug toxicity screening, or other therapeutic interventions (e.g., cellular implants) [27, 48, 49].

In this study, the first steps towards label-free identification of umbilical cord tissue and stem cell culturing were assessed. By measuring AF and SHG after TPM, we were able to visualize the major anatomical compartments of the human UC. A detailed description of these anatomic locations, their composition, and their potential stem cell content can be found elsewhere [2, 8, 13]. We clearly observed the cellular arrangement in the perivascular zones and vessel walls. In addition, we detected stromal cells within the less densely populated cord matrix and subamniotic zone. Our findings closely relate to a number of histological studies, which already described differences in radial distribution of stromal cells and extracellular matrix (ECM) components [39, 50]. Similar structures and cellular distribution patterns could be observed by our imaging approach, for example, the presence of tripolar cells in the subamniotic zone. Of note, our study visualized the cellular and structural arrangement throughout the cord using only the intrinsic fluorescence of cells and tissue components generated after pulsed laser excitation, without additional manipulation of the samples.

In contrast to a previous report by Uchugonova and König, who imaged different subpopulations of cells using a label-free setup [41], we could not discriminate between possible subpopulations of cells based on differences in autofluorescence signal intensity. Although AF was highly abundant in the walls of the umbilical vessels, individual cells did not show increased or decreased intensities compared to cells in other UC areas. Nevertheless, we conducted an additional experiment to stain for nuclei within the tissue to investigate the colocalization of cells and AF. Surprisingly, we observed a differential fluorescent intensity in DNA stain (DAPI). We assume that such discrepancy is suggestive of differences in DNA content between the smooth muscle cells and the adventitial cells, given that the staining efficiency is equal for the different cell types mentioned. Previous studies showed that the integrated fluorescence intensity of DAPI gives a good measure of DNA content [51, 52]. This is likely due to differences in metabolic state of the aforementioned cells, with smooth muscle cells being more active than the surrounding supportive cells. To further assess this finding, immunological staining but also ultrastructural analysis of the chromatin using transmission electron microscopy should be performed. Furthermore, others have documented the isolation of multipotent stem cells from the umbilical vein perivascular zone [53]. As such, it should be investigated whether DNA content correlates with the presence of multipotent progenitor cells, using, for example, the TPM/SHG imaging approach in conjunction with fluorescently labeled antibodies against multiple progenitor population-related markers (e.g., SSEA-4, CD271, and CD133).

To validate our imaging approach, we included the analysis of chondrogenic differentiated WJ-MSCs pellets. Rice et al. reported the quantitative use of two-photon excitation fluorescence and SHG for noninvasively monitoring MSCs differentiation [40]. They indicated that, by measuring the endogenous sources of contrast such as collagen, changes in cell metabolic activity, morphology, and extracellular matrix production can be visualized. As such, we could clearly detect the chondrogenic pellet and distinguish the newly formed matrix and cells scattered throughout the scaffold. Additionally, we imaged monolayer cultures of WJ-MSCs that were fully differentiated towards the adipogenic and osteogenic lineage. Two-photon excitation fluorescence and SHG imaging of adipo- and osteogenic differentiation were already reported for human bone-marrow-derived MSCs cultures [35, 40]. After adipogenic differentiation, we observed similar voids in the fluorescent signal due to lipid droplet accumulation. For the osteogenic differentiation, low SHG signal was detected, originating from collagen deposition in the ECM during differentiation. In this case, we did not expect dramatic signal changes, since our previous report indicated that WJ-MSCs represent an immature progenitor of* in vitro* osteogenesis [38]. Furthermore, our cultures were differentiated at normal oxygen levels, while Rice et al. observed increased collagen deposition under hypoxic differentiation conditions [40]. Nevertheless, our label-free and IHC analyses confirm the trilineage differentiation potential of our WJ-MSCs cultures.

Apart from imaging the UC tissue, we were able to visualize WJ-derived cells, both during isolation and in culture. While the cultured cells mainly presented without SHG, AF signal originated from the organelle rich perinuclear zone. Such finding is of interest since it could allow for the detection of cells based on their specific intracellular molecules. Using flow cytometry, Molinos et al. recently reported the detection of three distinct cell subsets based on a difference in autofluorescent signal [54]. Exploiting endogenous fluorophores as biomarkers for cell detection might be of beneficial use for veterinary research of the umbilical cord, since immunomarkers are not always available (e.g., equine or canine research) [31]. Further studies are required to validate different laser imaging setups (e.g., flow cytometry versus confocal laser scanning microscopy) and characterize the specific signals originating from the cells.

Besides the visualization of growing cells on coverslips, our AF and SHG based imaging approach is ideally suited to visualizing the initiation of explant cell cultures. Both the collagen rich explant fragments (SHG) and outgrowing cells (AF) were easily detected upon attachment to the culture chamber. Furthermore, the explant culturing technique preserves the initial WJ tissue (cellular niche) from which the cells arise. Consequently, we suggest that such label-free analysis can prove useful in discovering the origin of outgrowing cells, while imaging the explant culture process in real time.

It is currently unclear whether a specific tissue compartment of the UC contains multipotent stromal cells. Until now, it has not been possible to pinpoint a stem cell niche and subsequently follow the migration of the desired multipotent cells out of that tissue compartment. Their identification still occurs when cells are already in culture. Consequently, it still proves difficult to isolate specific populations of progenitors. A number of studies have attempted to address the* in situ* to* in vitro* transition of umbilical cord stem cells by correlating marker expression of cell cultures to their tissue origin [46, 55–57]. Yet, several issues further complicate such research, for example, the appearance of various cell populations with different isolation techniques (e.g., type I and type II cells) but also possible contamination by other cells and the lack of a specific multipotent stem cell biomarker, giving no conclusive results [50, 58]. Furthermore, for some markers, protein expression was induced or gradually diminished upon culturing, making it difficult to trace back the cells to their point of origin within the tissue [46]. We assessed the expression of multiple candidate stem cell markers for expression* in situ* and in culture. Initially, we assessed the expression of Oct4, a marker for pluripotent stem cells [44], *α*SMA, a marker commonly expressed by smooth muscle cells but also mesenchymal stromal cells [59], and ALDH1A1 which is expressed in both normal and cancer stem cell populations [45]. Using standard immunohistochemistry, we tried to localize differences in antigen expression patterns; however, we were not able to confine the markers to specific UC areas. Of note, in all cord compartments unstained cells could be observed, indicating that* in situ* differences in marker expression already preexist. Our findings are in line with previous reports, showing *α*SMA expression located in the umbilical vessels and WJ [60] and indicating the presence of Oct4 expressing cells within the WJ [43]. Additionally, we assessed the expression of vimentin, a cytoskeletal filament expressed by MSCs. Vimentin was abundantly expressed throughout the entire umbilical cord tissue except for the amniotic membrane. Our* in situ* analysis and previous reported cell culture data [38] confirm the recent report of Coskun and Can, in which they show that explants-derived cells originate from the UC stroma and not from the amniotic membrane as determined by their positive expression of vimentin and *α*SMA in culture [46].

In our explant cell cultures, a variable expression pattern was shown for *α*SMA and Oct4. In contrast to these markers, ALDH1A1 was expressed in all cultured cells. Furthermore, differences in ALDH1A1 intensity were observed between the smaller tripolar-shaped cells and the larger fibroblast-like cells. Whether those cells can be traced back to the tripolar cells in the subamniotic zone remains to be determined in ongoing experiments. Other studies already showed ALDH expression in primitive multipotent stem cells isolates [61–63]. Because of the differential expression pattern in umbilical cord tissue and isolated cells, more studies on the expression of this enzyme during culture initiation should be performed. Moreover, several stem cell related markers are difficult to trace back to their tissue of origin as their expression is influenced by both the isolation and the culture methods used (phenotype change) [46, 57, 64]. Cells derived from different compartments of the human umbilical cord were shown to express different amounts of CK subtypes in culture, depending on their isolation method [64]. We found expression of pan-CK at multiple sites in the umbilical cord, similar to the recently reported* in situ* study by Coskun and Can [46]. The pan-CK antibody used in this study, which contains CK 5, 6, 8, 17, and 19 subtypes, stained positive in all stromal cells and perivascular areas, but the highest staining intensity was observed for the amniotic epithelial cells lining the cord.

Other MSCs confined markers such as N-cadherin or desmin [56] are interesting candidates for tracking studies of explants in combination with the label-free imaging setup. We suggest that, by using AF and SHG imaging combined with specific antibody labeling, cellular outgrowth from the explants can be followed in real time (fluorescence lifetime imaging) with the potential to identify different cell subtypes. Such approach is already used in cancer research, imaging both cancer cell migration and ECM remodeling [65–67].

Collectively, this study shows that AF and SHG detection is a potent and easy approach for the visualization of stem cells* in situ* and may form a starting point for further biological studies of umbilical cord-derived stem cells. AF and SHG are optimally suited for visualization of live explants in culture. As such, the imaging approach can be a useful tool for assessing the* in situ* to culture transition of stem cells as well as for determining optimal isolation and culturing conditions. We speculate that AF and SHG imaging could prove useful in discovering the origin of outgrowing cells, while imaging the explant culture process in real time. Furthermore, the potential for high-resolution live imaging should be further explored in conjunction with other modern labeling techniques, such as antibodies conjugated to near-infrared excitable fluorophores [68, 69], quantum dots [70], or other nanoparticles [71, 72], provided there is no spectral overlap. Label-free monitoring of stem cells, in combination with such advanced staining techniques, opens perspectives for better cells characterization both* in situ* and* ex vivo*, by simultaneously visualizing resident cells and extracellular components. Hence, AF and SHG imaging can be a vital additional tool for unraveling the stem cell niche within the umbilical cord and other tissues.

## Supplementary Material

Correctly formatted supplementary information is supplied as attachment. short description: Figure S1: Negative control images of umbilical cord tissue staining for αSMA, Oct4, and ALDH1A1 expression; Figure S2: phase contrast image of WJ-MSCs in culture; Figure S3: Validation images and label-free imaging of adipogenic and osteogenic differentiation; Figure S4: images of umbilical cord tissue staining for vimentin and pan-CK expression and the respective negative controls.

## Abbreviations

*α*SMA: Alpha smooth muscle actin
AF: Autofluorescence
ALDH1A1: Aldehyde dehydrogenase family 1 member A1
DAB: Diaminobenzidine
DAPI: 4′,6-Diamidino-2-phenylindole
ECM: Extracellular matrix
HRP: Horseradish peroxidase
MSCs: Mesenchymal stem cells
NFR: Nuclear Fast Red
Oct4: Octamer-binding transcription factor 4
pan-CK: Pan cytokeratin
P/S: Penicillin-streptomycin
PBS: Phosphate-buffered saline
PFA: Paraformaldehyde
SHG: Second harmonic generation
TBS-T: Tris-buffered saline with 0.05% Tween-20
TPM: Two-photon confocal laser scanning microscopy
UC: Umbilical cord
WJ: Wharton's jelly
WJ-MSCs: Wharton's jelly-derived mesenchymal stem cells.
