# Supplementary material for: Label-Free Imaging of Umbilical Cord Tissue Morphology and Explant-Derived Cells
Source: Stem Cells Int. 2016 Sep 26;2016:5457132. doi: 10.1155/2016/5457132 (PMC5056264; doi:10.1155/2016/5457132)
Supplement: Supplementary file 1 — Correctly formatted supplementary information is supplied as attachment. short description: Figure S1: Negative control images of umbilical cord tissue staining for αSMA, Oct4, and ALDH1A1 expression; Figure S2: phase contrast image of WJ-MSCs in culture; Figure S3: Validation images and label-free imaging of adipogenic and osteogenic differentiation; Figure S4: images of umbilical cord tissue staining for vimentin and pan-CK expression and the respective negative controls. [file 5457132.f1.pdf]

Label-free imaging of umbilical cord tissue morphology and explant-derived cells

Raf Donders, Kathleen Sanen, Rik Paesen, Eli Slenders, Wilfried Gyselaers, Piet Stinissen, Marcel Ameloot and Niels Hellings

Supplemental figures

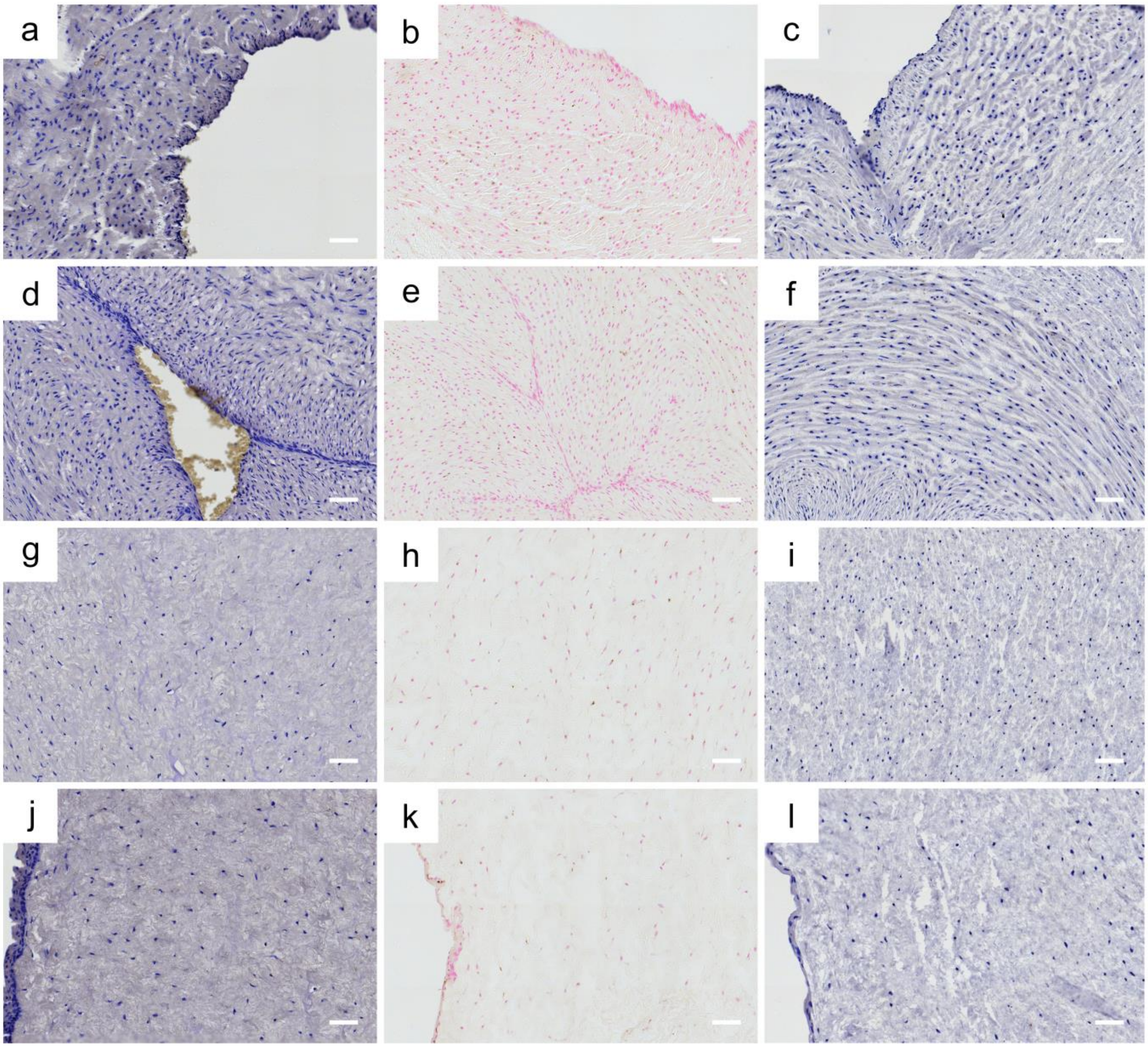

Fig. S1. Control immunohistochemical staining for αSMA (a, d, g & j), Oct4 (b, e, h & k) and ALDH1A1 (c, f, i & l) of umbilical cord areas: vein (a, b & c), arteries (d, e & f), Wharton’s jelly (g, h & i), and cord edge and amniotic epithelium (j, k & l). In a & d some residual HRP activity (brown) of remaining red blood cells is visible. Scale bars = 50 µm.

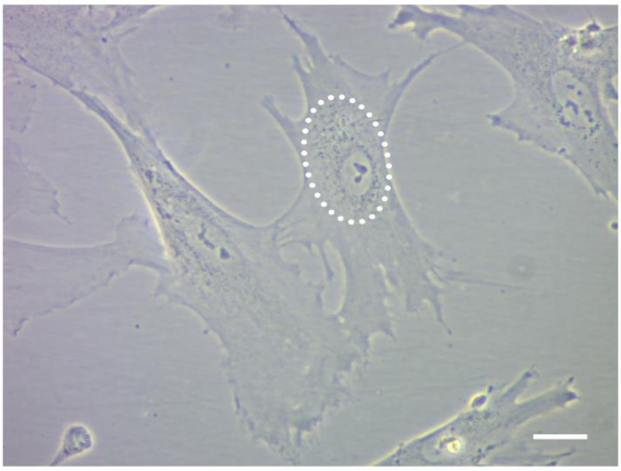

Fig. S2. Phase contrast image of WJ explant-derived cells displaying a fibroblast-like morphology in culture. The cells have large nuclei containing multiple nucleoli and a perinuclear area of organelles (white dotted line). Scale bar = 20 µm.

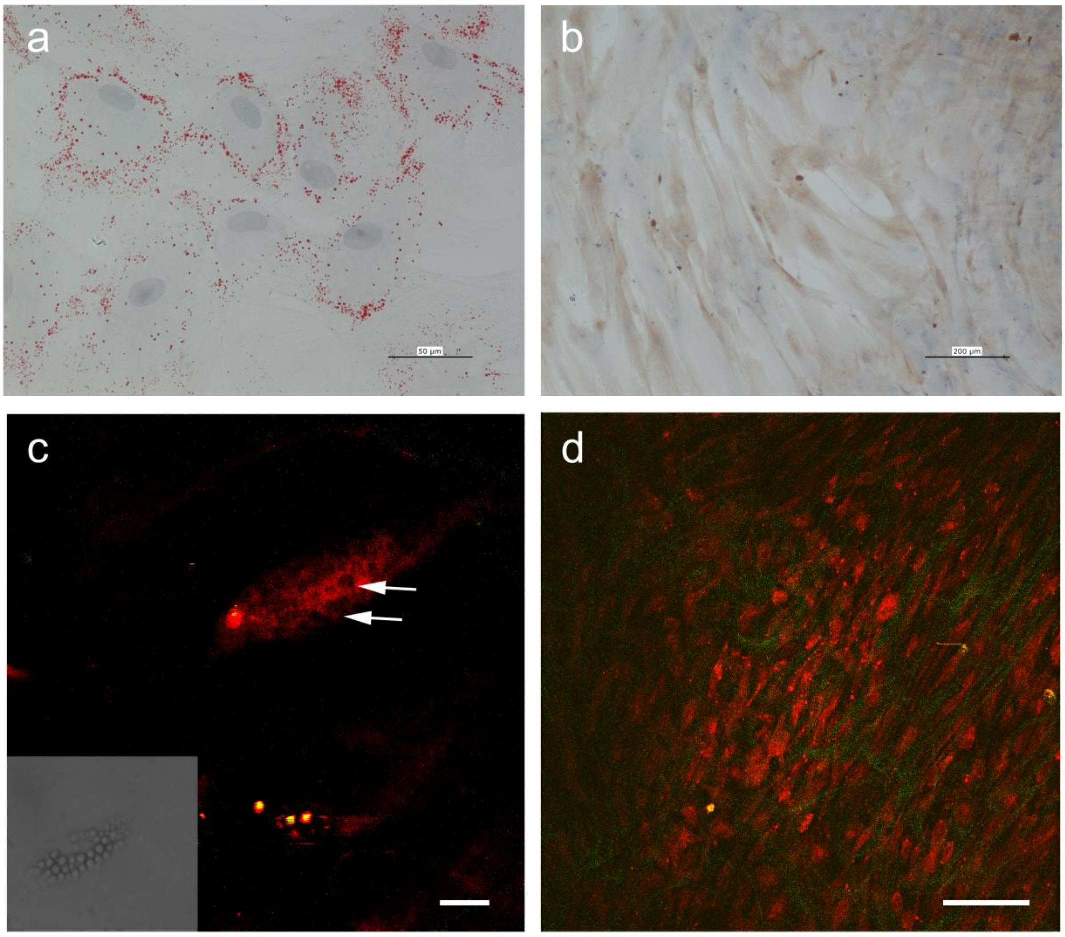

Fig. S3. TPM and SHG imaging of WJ-MSCs adipogenic and osteogenic differentiation. (a) oil red o staining of lipid accumulation in the cytoplasm, visualizing the droplets (red) and nucleus (blue), scale bar = 50 µm. (b) de novo expression of osteocalcine after osteogenic differentiation. Scale bar = 200µm. (c&d) AF (red) and SHG (green) imaging of (c) adipogenic and (d) osteogenic differentiation (scale bar = 200µm). (c) Arrows: lipid droplets cause voids in the fluorescent signal; Inset = brightfield image of the same cell. Scale bar = 20 µm. Representative images for 2 independent experiments are shown.

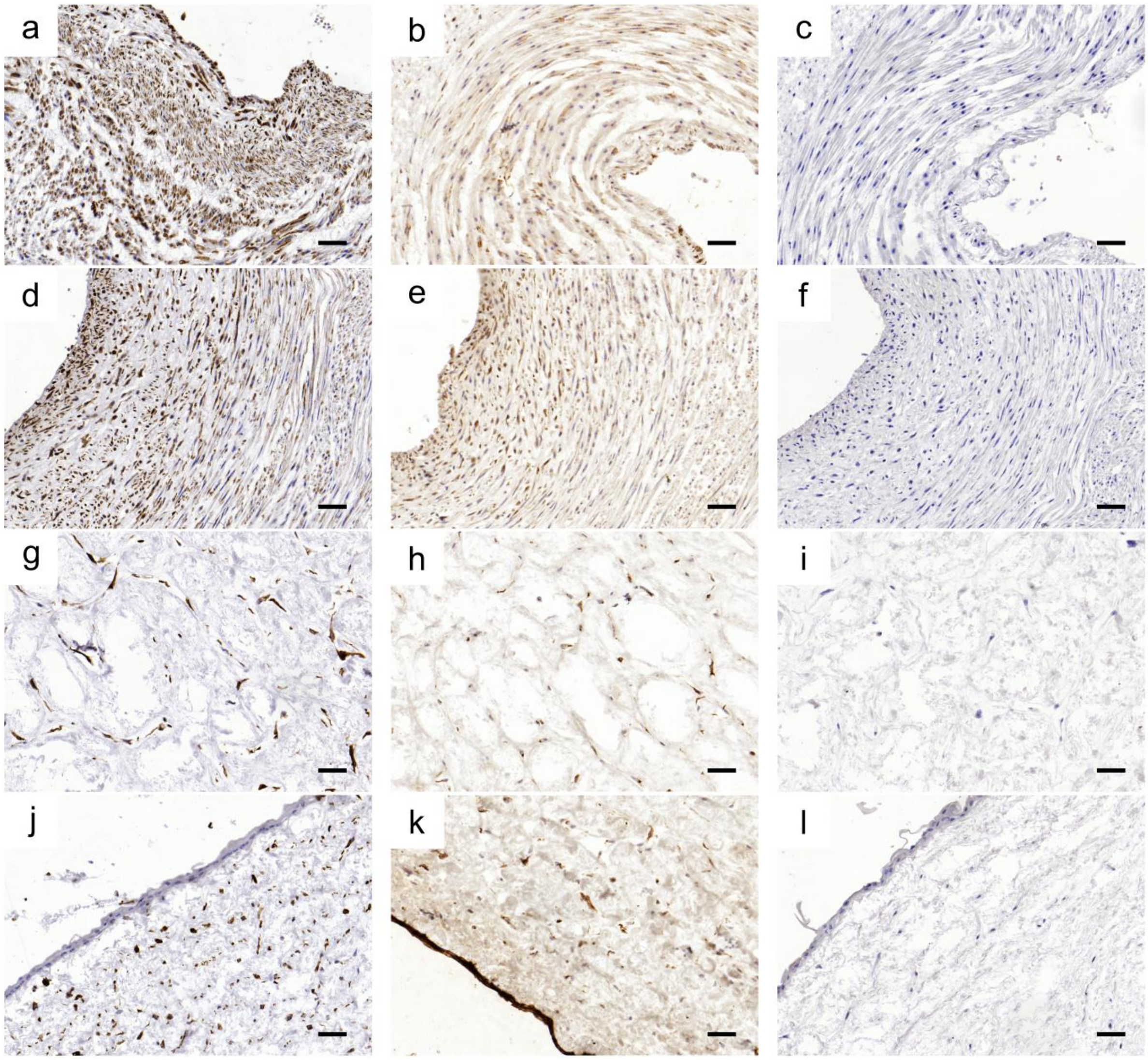

Fig. S4. Immunohistochemical staining for vimentin (a, d, g & j), pan-CK (b, e, h & k) and the respective control staining (blank; c, f, i & l) of umbilical cord areas: vein (a, b & c), arteries (d, e & f), Wharton's jelly (g, h & i), and cord edge and amniotic epithelium (j, k & l). Positive expression is depicted by brown coloring of the cell cytoplasm. Nuclei and cytoplasm were counterstained in blue using Mayer's hematoxylin. Scale bars = 50 µm. Representative images for 2 independent experiments are shown.
